# Community-Based Health Insurance Membership Renewal Rate and Associated Factors among Households in Gedeo Zone, Southern Ethiopia

**DOI:** 10.1155/2022/8479834

**Published:** 2022-10-03

**Authors:** Abdene Weya Kaso, Yofitahe Yohanis, Berhanu Gidisa Debela, Habtamu Endashaw Hareru

**Affiliations:** ^1^School of Public Health, College of Medicine and Health Science, Dilla University, Dilla, Ethiopia; ^2^Department of Public Health, College of Health Science, Arsi University, Asella, Ethiopia

## Abstract

**Background:**

Community-based health insurance (CBHI) scheme is an emerging strategy to achieve universal health coverage and protect communities in developing countries from catastrophic financial expenditure at the service delivery point. However, high membership discontinuation from the CBHI scheme remained the challenge to progress toward universal financial protection in resource-constrained countries. Therefore, this study assessed the community-based health insurance membership renewal rate and associated factors in the Gedeo zone, Southern Ethiopia.

**Methods:**

We conducted a community-based cross-sectional study among households in the Yirga Chafe district, Gedeo zone, Southern Ethiopia, from September 10 to 30, 2021. We used a multistage simple random sampling to recruit 537 respondents. We entered data into Epi-Info 7 and exported it to SPSS version 25 for analysis. We used a logistic regression model to determine factors associated with the CBHI scheme membership renewal. Variables with a *P* value of <0.05 and a 95% confidence level were considered to be significantly associated with the outcome variable.

**Results:**

We found the respondents' CBHI membership renewal rate was 82.68%. Those who enrolled in the CBHI scheme >3years (AOR = 3.12; 95% CI: 1.40–6.97), having illnesses in the last three months (AOR = 2.97; 95% CI: 1.47–5.99), the CBHI premium affordability (AOR = 12.64; 95% CI: 3.25–49.38), good knowledge of the CBHI scheme (AOR = 21.11; 95% CI: 10.63–41.93), perceived quality of health service (AOR = 4.21; 95% CI: 1.52–11.68), and favorable attitude towards the CBHI scheme (AOR = 3.89, 95% CI: 1.67–9.04) were significantly associated with the CBHI program membership renewal rate.

**Conclusion:**

In our study, we found the magnitude of CBHI members who discontinued their CBHI scheme membership was high. Besides, we found that the affordability of the CBHI premium, respondents' attitude, and knowledge of the CBHI program were predictor factors for dropout from the CBHI membership. Therefore, the government should consider the economic status of communities during setting the CBHI program contribution. Moreover, awareness creation through health education should be provided to improve participants' knowledge and perception of the CBHI program.

## 1. Introduction

In low- and middle-income countries (LMIC), out-of-pocket payments (OOP) were a means of financing health-care services for a decade [1]. Countries that rely too heavily on out-of-pocket payments to fund their health systems put a significant financial strain on households, pushing them to catastrophic health expenditures. Every year, around 200 million peoples worldwide face financial hardship as a result of OOP for health-care services [2, 3]. In Sub-Saharan Africa, out-of-pocket spending accounts for more than 40% of the total health-care expenditure, putting a significant financial strain on the poor [4]. The magnitude of OOP accounted for 34% of the total health-care expenditures in Ethiopia. As a result, a significant number of households were exposed to financial hardship for many years [5–7]. However, to bring the cost of catastrophic health care to insignificant levels, the World Health Organization (WHO) recommended the OOP payment at a level of less than 15 to 20% of the total health-care expenditure. In addition, the WHO declared the universal health coverage (UHC), a global initiative to give everyone access to affordable health-care services. Thus, the community-based health insurance (CBHI) scheme has been advocated as a viable strategy for protecting the poor in developing countries from the catastrophic burden of financing health services [8, 9]. The introduction of the CBHI scheme in health-care financing of LMIC improved health-care-seeking behavior, availability of drugs, and the quality of services through mobilized resources in public health facilities [10–13]. Despite the plan of universal financial protection, there is variation in households' enrollment rate in the CBHI scheme from country to country. The willingness to participate in the CBHI scheme was 86.7% in Bangladesh [14], 69.6% in Saudi Arabia [15], 59.4% in Nigeria [16], and 46% in Cameron [17]. In Ethiopia, since the CBHI program was launched in the health financing reforms, a significant number of households were enrolled in the CBHI scheme, with variation in enrolment rate from region to region [10, 18]. For instance, studies carried out in the Jimma zone [19] and Bench Maji zone [20], West Shoa zone [21], and Gojjam zone [22] revealed that the willingness to join (WTJ) the CBHI scheme was 78%, 71%, and 81.5%, respectively. In addition, WTJ for this voluntary health insurance was 73% in West Arsi zone [23] and 78.5% in Buno Bedele zone [24]. Moreover, the willingness to pay for the CBHI program was 74.8% in Gemmachis district [25], 80% in Fogera district [26], and 79% in EfratanaGedem districts of the Amhara region [27]. However, the capacity of the CBHI scheme to retain its members is crucial to its long-term viability, and a high proportion of the CBHI scheme membership discontinuation remained the challenge to progress toward universal financial protection in LMIC [28]. The report from Bangladesh revealed that about 23% of the CBHI members renewed their CBHI memberships over the initial three years of operation [29]. A study in Uganda revealed that 25.1% of the rural households in the CBHI scheme were dropped out from the program [13]. In addition, a study in Burkina Faso indicates that rural households' dropout rate from the CBHI scheme ranges from 30.9% to 45.7% [30]. Besides, in Ethiopia, high dropout rates during the period of membership renewal were a great challenge to attaining the proposed coverage of 80% by 2020 [18]. The dropout rate was 31.9% in the Manna district [31], whereas it increased to 37.3% in the Dera district [32].

The synthesis of previous studies showed that the CBHI scheme membership renewal is influenced by the affordability of CBHI premium, quality of health care, households' attitudes, and knowledge of the CBHI scheme, availability of basic logistics and supplies, and trust of households' towards premium management [33–37]. There have been several studies on willingness to join and pay for the CBHI scheme; however, few have offered insight on the CBHI scheme membership renewal, and little is known about the factors that influence decisions to renew the health insurance schemes. Also due to the lack of the ability to pay for the scheme contribution, the evidence regarding the households' CBHI membership renewal after the current CBHI contribution revision is limited. Studies on the CBHI membership renewal rate can provide information on the design and implementation of the intervention that assists the sustainability of an insurance scheme. Therefore, this study assessed the household's CBHI membership renewal rate and associated factors among rural communities in the Yirga Chafe district, Gedeo zone, Southern Ethiopia.

## 2. Method and Material

### 2.1. Study Setting, Design, and Population

We conducted a community-based cross-sectional study design from September 10 to 30, 2021, in the Yirga Chafe district, Gedeo zone, Southern Ethiopia. The district comprises 27 kebele (the smallest administrative unit) with an estimated population of 249,599 and 49,819 households. It is 38 kilometers far away from Dilla, the Zonal town of the Gedeo zone. All rural households in the Yirga Chafe district who were members of the CBHI scheme were considered source populations and households in the randomly selected kebele were study populations. A household who was a member of the CBHI scheme for more than one year, aged greater than or equal to 18 years old, and working in the informal sectors was included in the study. Respondents who were critically ill and unable to participate in an interview during the data collection period were excluded from the study.

### 2.2. Sample Size and Sampling Procedure

We calculate the sample size using the single population proportion formula with the assumption of the magnitude of household CBHI program membership renewal (68.1%) [31], a confidence level of 95%, 1.5 design effects, and a 5% margin of error. The final calculated sample size was 551 after adding a 10% nonresponse rate. We used a multistage random sampling technique and selected eight kebele using a simple random sampling technique. The total samples were proportionally allocated to each selected kebele based on the respective number of a household enrolled in the CBHI. Households were our main sampling unit, and we considered heads of the households primarily for interviews from the selected households using a systematic random sampling method. For household heads that were not available, adults aged 18 years old and who could properly respond to the questions were interviewed. In case, more than one adult was available in the households, one adult was selected randomly and interviewed. If the households were not available during the visit, the next visit was rearranged.

### 2.3. Data Collection Tool and Technique

We collected data using a structured questionnaire, which was initially prepared in English and translated into the local language, Giduaffa, and retranslated back into English. The questionnaire consists of five parts: socio-demographic characteristics, health and health-care-seeking behavior, health service quality perception, knowledge of the CBHI scheme, and attitude towards the CBHI scheme. We pretested 5% of the questionnaires before the actual data collection period in a nearby kebele which is not included in the study. We modified our tool for clarity, validity, and logical consistency based on pretest findings. We assigned data collectors from 4th year public health officer students and trained them for two days on the objective of the study, the contents of the questionnaire, ethical issues, and the right of the respondents. The principal investigator coordinated the data collection and checked the collected questionnaires for consistency on daily basis.

### 2.4. Variables and Operational Definition

#### 2.4.1. Study Variables

Community-based health insurance membership renewal was the dependent variable, whereas various factors such as socio-demographic and economic-related factors (age, sex, marital status, occupation, educational status, family size, and income), health and health-care utilization-related factors (self-rated health status, illness experience in the last three months, history of chronic illness, and distance to the nearest health facility) and perception-related factors (knowledge about CBHI, attitude toward CBHI, and perceived quality of service), CBHI premium affordability, and length of enrolment in the CBHI program were the independent variables (Table 1).

#### 2.4.2. Operational Definition

CBHI membership renewal is acceptance of the CBHI scheme by the CBHI member to use and pay a premium for the next year and possess an updated service card.

### 2.5. Knowledge of CBHI

This refers to household heads' knowledge of the CBHI benefit package, its principles, and the route of access during seeking services. It was assessed using 13 yes or no items, and the “Yes” answer was the correct response and labelled as “1,” whereas the “No” answer was considered the wrong response and labelled as “0.” The scores of the knowledge item questions were computed, and the scores of the knowledge item questions were computed, and the knowledge score was categorized using Bloom's cut-off point, as poor (≤60%; less than 8 scores), moderate (61%–79%; 8–10 score), and high (80%–100%; 11 and above score). Subsequently, we grouped moderate and high knowledge into one category as good knowledge of the CBHI scheme.

### 2.6. Perceived Quality of Health Service

We measured the overall quality of health-care services using the availability of medical equipment and prescribed drugs, waiting time to get the card and observe health-care providers, waiting time between services, and health-care providers' friendliness. We measured this variable on a 5 Likert scale using a 5-point response from very poor to very good, and the internal consistency of the items was checked using the pilot study (Cronbach's alpha (*α*) = 0.78). We asked the respondents to rate the extent to which they perceived a set of seven questions regarding the health services they received from the nearby health centers contracted by the CBHI scheme. The composite score ranged from 7 to 35, and the health-care quality index was categorized using Bloom's cut-off point, as poor (less than 60%; less than 21 scores), medium (60%–79%; 21–27 score), and good (80%–100%; 28 and above score).

### 2.7. Attitude towards CBHI Scheme

The respondents' attitude towards the CBHI scheme was assessed using six internally consistent and reliable items (*α* = 0.732) ranging from strongly agree to strongly disagree. Bloom's cut-off point was used to categorize the attitude into negative (≤60%; <19), neutral (61%-79%, 19–23), and positive (80%-100%, ≥24–30). Subsequently, we grouped neutral and positive attitudes into one category tagged favorable, whereas negative attitudes were considered unfavorable attitudes towards the CBHI scheme.

### 2.8. Data Analysis

Data were entered into Epi-info 7 and exported to SPSS version 25 for analysis. We described categorical variables using frequency and percentage, whereas continuous variables were summarized by mean with standard deviation. Data were presented using tables and graphs. Multivariate logistic regression analysis was performed to identify the potential predictors of the CBHI membership renewal. We checked the model fitness by Hosmer-Lemeshow's goodness of fit test and sample adequacy test before the regression analysis. In addition, we determined the multicollinearity among independent variables by estimating the variance inflation factor. If a strong correlation was observed between two independent variables, we eliminated one of the two to avoid multicollinearity. All variables with a *P* value less than or equal to 0.25 in the bivariate logistic regression analysis were entered into a multivariate logistic regression model, and a *P* value of less than 0.05 and an adjusted odds ratio (AOR) with 95% CI were used to declare the predictors of the outcome variable.

## 3. Result

### 3.1. Socio-Demographic Characteristics of the Respondents

The response rate of our study was 97.5%. Out of the 537 respondents, the majority (81.8%) of them were males. The mean age of the participants was 36.01 years (SD = 8.66) and ranged from 21 to 59 years old. The majority (55.3%) of the respondents were protestant religious followers, (40.2%) had informal education, 366 (68.2%) were farmers, and 386 (71.9%) had a family size of less than five (Table 2).

### 3.2. Health Status and Health-Care-Seeking Behavior of Respondents

Almost more than two-fifths (48.8%) of the study respondents rated their health status as good. Only 74 (13.8%) of participants had reported the presence of chronic illnesses in their family. Two hundred seventy-six (51.4%) of them reported that their family members have encountered illnesses in the last 3 months. Out of 276 individuals who were ill, 274 (99.3%) sought and got medical care, of which 56.6% preferred to visit government health centers for the recent episode. The majority (87.7%) of the study participants travelled 30 or above minutes to get health services from the nearest health facility (Table 3).

### 3.3. Knowledge of the Study Participants regarding CBHI

We assessed the knowledge of participants regarding the benefits package and principles of the CBHI scheme and the route of accessing public health facilities as well as their knowledge of the CBHI premium. Among 537 participants' knowledge assessed, 392 (73%) know the principle of the CBHI scheme, 331 (61.6%) know the routes of accessing public health facilities for health-care services, and 491 (91.4%) know why they pay for CBHI program premium. Regarding the overall CBHI program knowledge, 438 (81.6%) of the respondents had good knowledge, whereas 99 (18.4%) of them had poor knowledge about the CBHI scheme (Table 4).

### 3.4. Respondents' Attitude toward the Community-Based Health Insurance Scheme

In our study, 434 (80.8%) respondents had a perception of the potential ability of the CBHI scheme to make health-care services more affordable. In addition, 411 (76.5%) of participants perceived that the CBHI improves households' health-care-seeking behavior, whereas 421 (78.4%) perceived that the CBHI program has the potential of increasing access to affordable health-care services. Besides, 384 (71.5%) and 374 (69.6%) individuals perceived that the CBHI scheme improves the availability of the drug at facilities and the quality of health services provided, respectively. Overall, more than three-fourth (78.4%) of participants had a favorable attitude toward the CBHI scheme (Table 5).

### 3.5. Perceived Quality of Health Service Delivery among CBHI Scheme Users

Our study showed that 332 (61.8%) of the respondents had a perception of the waiting time to get the card during getting service from public health facilities as good. More than two-thirds (70.6%) of the participants were rated the availability of laboratory services as good. In addition, 70.6% and 61.1% of respondents perceived that the waiting time between services and seeing a medical service provider was good (Table 6).

### 3.6. Respondents' Magnitude of Renewal of Community-Based Health Insurance Service

**Figure 1:**
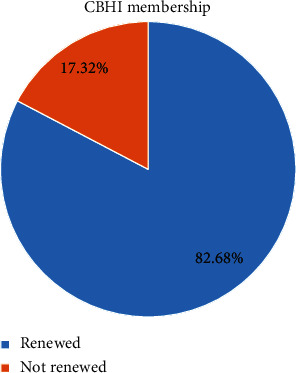
Community-based health insurance membership renewal rate among respondents in Yirga Chafe district, Southern Ethiopia, 2022.

**Figure 2:**
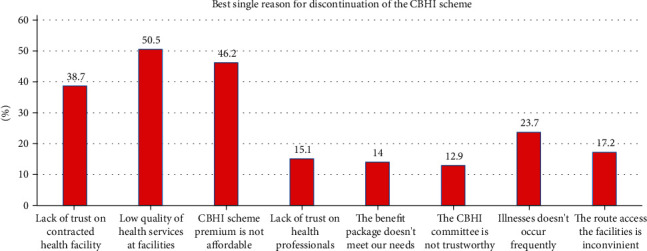
Respondents' reasons for dropout from CBHI scheme membership in Yirga Chafe district, Southern Ethiopia, 2022.

In our study, the magnitude of the CBHI membership renewal rate among respondents was 82.68% [95% CI: 79.47–85.89] (Figure 1). Among 93 (17.32%) respondents who did not renew their CBHI membership, more than half (50.5%) described the poor quality of health service provided in the public health facilities as the main reason for discontinuation of the CBHI scheme. In addition, 46.2% and 38.7% of respondents' reason out that the CBHI premium unaffordability and lack of trust in the capacity of contracted health facilities to provide all health-care services were factors for discontinuation of the CBHI scheme membership (Figure 2).

### 3.7. Factors Associated with Respondents' CBHI Scheme Membership Renewal

In multivariate logistic regression analyses, the odds of the CBHI membership renewal were associated with the presence of illnesses in three months, length of enrollment in the CBHI scheme, affordability of the CBHI premium, perception of quality of service, respondents' attitudes, and knowledge of the CBHI scheme. Accordingly, having illnesses in the last three months was almost three (AOR = 2.97; 95% CI: 1.47, 5.99) times higher odds of the CBHI membership renewal compared to their counterparts. The odds of renewing the CBHI membership among participants who enrolled in the scheme for 3 and above years were almost 3 times higher compared to those who participated in the CBHI for less than 3 years (AOR: 3.12, 95%CI = 1.40, 6.97). Besides, respondents who perceived the CBHI premium as affordable had 12.64 times higher odds of the CBHI membership renewal as compared with those who perceived the CBHI premium as unaffordable (AOR = 12.64, 95% CI: 3.25–49.38). The odds of the CBHI membership renewal among respondents with good knowledge and a favorable attitude towards the CBHI scheme were around 21 and 4 times higher than those with poor knowledge and favorable attitude toward the CBHI scheme (AOR = 21.11, 95% CI: 10.63–41.93) and (AOR = 3.89, 95% CI: 1.67–9.04), respectively. Besides, respondents who perceived health service quality as good were 4.21 times more likely to renew their CBHI scheme membership compared to their counterparts (AOR: 4.21, 95%CI = 1.52, 11.68) (Table 7).

## 4. Discussion

Community-based health insurance plays a great role in reducing catastrophic health-care expenditure and increasing the utilization of the health-care service and the progress towards achieving UHC. However, households' dropout from CBHI scheme membership was a challenge to increase the CBHI enrolment coverage to 80% by 2020 [1, 8, 18]. Therefore, this study assessed households' CBHI scheme membership renewal rate and associated factors in the Yirga Chafe district, Gedeo zone, Southern Ethiopia. We found the magnitude of the CBHI membership renewal rate of 82.68%, which is similar to the reports from Vietnam, 78.9% [38], and Ethiopia, 82% [39], and higher than study findings conducted in Bangladesh, 38% [29]; Burkina Faso, 45.7% [30]; Ghana, 34.8% [40]; Dera district, Ethiopia, 62.7% [32]; and Jimma zone, 68.1% [31]. This is explained by the difference in the study populations' socio-demographic and economic characteristics and the study period. Besides, some studies described the magnitude of CBHI scheme membership over the years.

Our study revealed that respondents who encountered illnesses in the last three months were more likely to renew the CBHI scheme membership compared to their counterparts. This finding is in line with the findings of studies conducted in Tanzania [41], Ghana [40], and Ethiopia [28, 42]. This could be due to the catastrophic health-care expenditure faced by family members who were sick and needed medical attention. Moreover, the quality of health-care service provided was another key factor to determine the respondents' CBHI scheme membership renewal. We found that respondents who perceived the quality of health-care service was good had higher odds of the CBHI membership renewal rate as compared to those who perceived poor. The same finding was reported from studies done in Ghana [40], Tanzania [41], Rwanda [35], and Ethiopia [36, 43, 44]. This is explained by individuals who had perceived good quality of health-care services might get drugs and diagnostic services in the facilities and observed health providers in short waiting times which increase their satisfaction to retain their CBHI scheme membership.

The respondent's attitude toward the importance of the CBHI scheme had also a greater effect to renew the CBHI scheme membership. We found that individuals' renewal of the CBHI membership was positively associated with a favorable attitude towards the importance of the CBHI program. This finding is in agreement with other studies done in Uganda [45] and Ethiopia [46]. In addition, we found that the CBHI membership renewal was positively associated with respondents' knowledge of the CBHI program benefit packages and principles. Similar findings were also reported from studies done in Uganda [45], Gemmachis district, Ethiopia [25], Dera district, Ethiopia [32], and Ethiopia [39, 44]. This could be because individuals who had good knowledge of the CBHI scheme might have a better understanding of the principles and benefits package of the CBHI scheme and easily understand the benefits of renewing their health insurance scheme membership.

Our findings demonstrate that the CBHI membership renewal was significantly associated with the affordability of the CBHI scheme premiums. This finding was consistent with other studies in Rwanda [35], Ghana [40], and Ethiopia [43, 44, 47, 48]. This might be explained by if the CBHI scheme contribution premium was affordable; the low-income earners can pay for the CBHI scheme premium and are more likely to retain their CBHI program membership. In addition, we found the length of enrollment is a crucial factor that significantly determines the respondents' CBHI program membership renewal. Compared to respondents who enrolled in the CBHI program for less than 3 years, ≥3 years of enrollment in the CBHI scheme is associated with around 3 times more likely to renew their membership. This result is in line with the findings of studies conducted in India [49] and Ethiopia [32] that found that individuals who had a longer length of enrollment in the CBHI program were more satisfied and retain their membership than their counterparts. This might be explained by individuals who participated in the CBHI members for a long time had increased awareness of the importance of the CBHI program that reduces their discontinuation of the CBHI membership. Even though we provided scientific information for the sustainability of the existing CBHI scheme in Ethiopia, our study had few drawbacks. First, it was conducted in a single study area, and the sample size is small. Second, it has the limitation of generalizability as we assessed the magnitude of the CBHI scheme renewal rate among rural communities and excluded households residing in urban areas.

## 5. Conclusion

In our study, we found the magnitude of CBHI members who discontinued their CBHI scheme membership was high. Besides, we found that the affordability of the CBHI premium and respondents' attitudes and the knowledge of the CBHI program were predictor factors for dropout from the CBHI membership. Therefore, the government should consider the economic status of communities during setting the CBHI program contribution. Moreover, awareness creation through health education should be provided to improve participants' knowledge and perception of the CBHI program.

## Figures and Tables

**Table 1:** Description and measurement of the variable used in the logistic regression model.

S.no	Variables	Description	Measurement
Dependent variable			
1	CBHI renewal	Households' renewal of the CBHI membership	1 if renewed; 0 if dropped

Independent variables			
2	Age	Age of respondents	0 if18-30 years; 1 if 31-45 years; and 2 if 46 and above years
3	Sex	Gender of respondents	1 if female; 0 if male
4	Marital status	Respondents	1 if married; 0 if otherwise
5	Family size	Household size of the respondent	0 if less than or equal to five; 1 if greater than 5
6	Monthly income	Average monthly income of respondent	O if less than 1000 birr; 1 if 1,000-2,000 birr; 2 if above 2000 birr
7	Educational status	Educational status of respondents	0 if nonformal; 1 if primary education (1–8); and 2 if secondary and above
8	Occupational status	Occupational status of participants	0 if farmer; 1 if merchant; and 2 if other
9	Time to access health facilities	The time it takes the participant to reach health facilities	1 if less than 30 minutes; 0 if greater than 30 minute
10	Length of enrollment	Respondents' length of enrollment in the CBHI scheme	0 if enrolled <3 years; 1 if enrolled >3 years
11	Illness in last 3 months	Presence of illness in the households in the last 3 months	1 if yes; 0 if no
12	Overall knowledge	Respondent's awareness of the CBHI scheme	1 if good knowledge; 0 if poor knowledge
13	Overall attitude	Respondents' attitudes toward the CBHI program	1 if favorable attitude; 0 if unfavorable
14	CBHI premium	Affordability of the CBHI contribution	1 if affordable; 0 if not affordable
15	Perceived quality service	Respondent's perception of the quality of services they received	1 if good; 2 if medium; and 3 if poor

**Table 2:** Sociodemographic characteristics of respondents in South Central, Ethiopia, 2022.

Variables	Categories	Frequency (%)
Sex	Male	439 (81.8)
	Female	98 (18.2)

Age (in years)	18-30	156 (29.1)
	31-45	303 (56.4)
	46 and above	78 (14.5)
	Mean (SD)	36.01 (8.66)

Marital status	Married	470 (87.5)
	Others^a^	67 (12.5)

Religion	Muslim	79 (14.7)
	Protestant	297 (55.3)
	Orthodox	161 (30.0)

Education	Informal education	216 (40.2)
	Primary (1–8)	213 (39.7)
	Secondary and above	108 (20.1)

Ethnicity	Oromo	47 (8.8)
	Gedeo	476 (88.6)
	Amhara	14 (2.6)

Occupation	Farmer	366 (68.2)
	Merchants	85 (15.8)
	Others^b^	86 (16.0)

Monthly income	<1000 birr	137 (25.5)
	1000-2000 birr	317 (59.0)
	Above 2000 birr	83 (15.5)

Family size	<5	386 (71.9)
	≥5	151 (28.1)

Note: ^a^ = widowed/divorced, ^b^ = daily labor, housewife.

**Table 3:** Health and health-related characteristics of respondents in Gedeo zone, 2022.

Variables	Categories	Frequency (%)
Self-reported health status of households	Poor	72 (13.4)
	Medium	203 (37.8)
	Good	262 (48.8)

Chronic illness in the family	Yes	74 (13.8)
	No	463 (86.2)

Family members encountered illness in the last three months	Yes	276 (51.4)
	No	261 (48.6)

Seek treatment for the recent episode	Yes	274 (99.3)
	No	2 (0.7)

Frequency of visiting this health facility per year	≤3	301 (56.10
	>3	236 (43.9)

Place of treatment	Government health center	155 (56.6%)
	Government hospital	109 (39.8%)
	Private clinic	10 (3.6)

Time to reach health facility (in minutes)	<30 min	66 (12.3)
	≥30 min	471 (87.7)

**Table 4:** Respondents' knowledge of a community-based health insurance scheme.

Variables	Yes (%)	No (%)
Know the principle of CBHI	392 (73.0)	145 (27.0)
Know the route access to health facilities	331 (61.6)	206 (38.4)
CBHI is a good way of getting health expenditure	338 (62.9)	199 (37.1)
CBHI covers only services from public health facilities	523 (97.4)	14 (2.6)
CBHI covers only care within the country	449 (83.6)	88 (16.4)
CBHI does not cover transportation fee	530 (98.7)	7 (1.3)
CBHI covers outpatient care	514 (95.7)	23 (4.3)
CBHI covers inpatient care	512 (95.3)	25 (4.7)
CBHI does not cover medical care for cosmetic values	238 (44.3)	299 (55.7)
CBHI does not cover medical care for dental service	246 (45.8)	291 (54.2)
You pay CBHI premiums to finance your future health-care needs	491 (91.4)	46 (8.6)
CBHI scheme is not like a savings scheme, you will not receive interest and get your money back	481 (89.6)	56 (10.4)
If you do not make claims through CBHI, your premium will not be returned	528 (98.3)	9 (1.7)

Overall knowledge		
Poor	99 (18.4)	
Good	438 (81.6)	

**Table 5:** Respondents' perception towards community-based health insurance scheme, 2022.

Variables	Strongly disagree (%)	Disagree (%)	Neutral (%)	Agree (%)	Strongly agree (%)
CBHI makes health care affordable	0 (0%)	70 (13)	33 (6.1)	364 (67.8)	70 (13)
CBHI increases access to affordable health care	0 (0%)	74 (13.4)	42 (7.8)	347 (64.6)	74 (13.8)
CBHI improves household health-seeking behavior	0 (0%)	88 (16.4)	38 (7.1)	402 (74.9)	9 (1.7)
CBHI improves the quality of services provided	18 (3.4)	98 (18.2)	47 (8.8)	374 (69.6)	0 (0)
CBHI improves the availability of drugs at facilities	35 (6.5)	96 (17.9)	22 (4.1)	316 (58.8)	68 (12.7)
CBHI committee manages pooled funds very well	75 (14)	24 (4.5)	365 (68)	0 (0)	73 (13.6)

Overall attitude	Unfavorable	116 (21.6)			
	Favorable	421 (78.4)			

**Table 6:** Community-based health insurance members' perception of the quality of health services, 2022.

Variables	Very poor (%)	Poor (%)	Neutral (%)	Good (%)	Very good (%)
Waiting time to get the card	3 (0.6)	91 (16.9)	36 (6.7)	332 (61.8)	75 (14)
Availability of laboratory service	19 (3.5)	94 (17.5)	34 (6.3)	379 (70.6)	11 (2)
Waiting time to see a medical provider	13 (2.4)	92 (17.1)	29 (5.4)	328 (61.1)	75 (14%)
Waiting time between service	28 (5.2)	86 (16)	33 (6.1)	379 (70.6)	11 (2)
Health-care provider friendly	5 (0.9)	78 (14.5)	38 (7.1)	339 (63.1)	77 (14.3)

Perceived quality of health service					
Poor	104 (19.4)				
Medium	93 (17.3)				
Good	340 (63.3)				

**Table 7:** Factors associated with respondents' community-based health insurance membership renewal in Yirga Chafe district, Southern Ethiopia, 2022.

Categories	Renewed (%)	Dropped (%)	COR (95% CI)	AOR (95% CI)
*Age categories*				
18-30 years	125 (28.2)	31 (33.3)	1	1
31-45 years	254 (57.2)	49 (52.7)	1.29 (.781, 2.12)	1.42 (.389, 5.08)
46 and above years	65 (14.6)	13 (14)	1.24 (0.607, 2.53)	1.33 (0.421, 4.20)

*Marital status*				
Married	385 (86.7)	85 (91.4)	0.62 (0.283, 1.33)	0.93 (0.226, 3.83)
Other	59 (13.3)	8 (8.6)	1	1

*Sex*				
Male	358 (80.6)	81 (87.1)	1	1
Female	86 (19.4)	12 (12.9)	1.62 (0.846, 3.11)	1.51 (0.558, 4.07)

*Educational status*				
Nonformal	171 (38.5)	45 (48.4)	1	1
Primary	180 (40.6)	33 (35.5)	1.44 (0.874, 2.36)	0.51 (0.220, 1.18)
Secondary and above	93 (20.9)	15 (16.1)	1.63 (0.863, 3.09)	0.78 (0.296, 2.03)

*Occupational status*				
Farmer	301 (67.8)	65 (69.9)	1	1
Merchant	67 (15.1)	18 (19.4)	0.81 (0.448, 1.44)	0.51 (0.162, 1.62)
Other	76 (17.1)	10 (10.7)	1.64 (0.805, 3.35)	1.97 (0.713, 5.46)

*Monthly income*				
Less than 1000 birr	98 (22.1)	39 (41.9)	1	1
1000-2000 birr	279 (62.8)	38 (40.9)	2.92 (1.77, 4.83)	0.51 (0.136, 1.90)
Above 2000 birr	67 (15.1)	16 (17.2)	1.67 (0.862, 3.22)	0.43 (0.08, 2.42)

*Time to access health facilities*				
< 30 minutes	57 (12.8)	9 (9.7)	1.38 (0.655, 2.89)	1.9 0(0.642, 5.64)
≥ 30 minutes	387 (87.2)	84 (90.3)	1	1

*Length of enrollment*				
<3 years	182 (41.0)	68 (73.1)	1	1
≥3 years	262 (59.0)	25 (26.9)	3.88 (2.36, 6.37)	3.12 (1.40, 6.97)^∗^

*Family size*				
≤ 5	310 (69.8)	76 (81.7)	1	1
> 5	134 (30.2)	17 (18.3)	1.93 (1.10, 3.40)	1.95 (0.762, 5.99)

*Illness in last 3 months*				
Yes	250 (56.3)	26 (28)	3.32 (2.04, 5.42)	2.97 (1.47, 5.99)^∗^
No	194 (43.7)	67 (72)	1	1

*Overall knowledge*				
Poor	34 (7.7)	65 (69.9)	1	1
Good	410 (92.3)	28 (30.1)	27.99 (15.92, 49.23)	21.11 (10.63, 41.93)^∗^

*Perceived quality service*				
Poor	59 (13.3)	45 (48.4)	1	1
Medium	79 (17.8)	14 (15.0)	4.04 (2.02, 8.10)	2.67 (0.940, 7.59)
Good	306 (68.9)	34 (36.6)	10.38 (5.34, 20.16)	4.21 (1.52, 11.68)^∗^

*Overall attitude*				
Unfavourable	69 (15.5)	47 (50.5)	1	1
Favourable	375 (84.5)	46 (49.5)	5.55 (3.43, 8.98)	3.89 (1.67, 9.04)^∗^

*CBHI premium*				
Not affordable	60 (13.5)	41 (44.1)	1	1
Affordable	384 (86.5)	52 (55.9)	5.05 (3.09, 8.25)	12.64 (3.25, 49.38^∗^)

## Data Availability

The datasets used or analyzed during this study were available from the corresponding author on reasonable request.

## Abbreviations

AOR: Adjusted odds ratio
CBHI: Community-based health insurance
CI: Confidence interval
COR: Crude odds ratio
LMIC: Low- to medium-income countries
OR: Odds ratio
UHC: Universal health coverage
OOP: Out of pocket
WHO: World Health Organization.
